# *Streptococcus* and related oral taxa are differentially abundant in patients with abdominal aortic aneurysms compared with atherosclerotic disease controls

**DOI:** 10.3389/fcvm.2026.1884994

**Published:** 2026-08-28

**Authors:** Joshua T. Geiger, Ann L. Gill, Mario Matabele, Joel Kruger, Baqir Kedwai, Daniel Lehane, Benjamin Ford, Michael C. Stoner, Michael B. Sohn, Steven R. Gill, Doran Mix

**Affiliations:** 1 Division of Vascular Surgery, University of Rochester Medical Center, Rochester, NY, United States; 2 Department of Microbiology and Immunology, University of Rochester Medical Center, Rochester, NY, United States; 3 Department of Biostatistics and Computational Biology, University of Rochester Medical Center, Rochester, NY, United States

**Keywords:** abdominal aortic aneursym, atheroscelorsis, inflammation, oral microbiome, *streptococcus*, vascular disease

## Abstract

**Objective:**

Bacterial DNA from oral microorganisms has been found in various cardiovascular tissues, including abdominal aortic aneurysm (AAA) tissue. In addition, experiments in murine models have shown that the oral pathogen *Porphyromonas gingivalis* can independently contribute to aneurysm formation. However, whether the oral microbial community differs between patients with AAA and those with advanced atherosclerotic disease remains unknown. This study tests the hypothesis that specific bacterial taxa are differentially abundant in patients with AAA compared to those with advanced atherosclerotic disease.

**Methods:**

A total of 73 oral wash samples were collected from 45 subjects with AAA and 28 with atherosclerotic disease. Bacterial DNA was isolated, and the V1-V3 segment of the bacterial 16S rRNA gene was amplified. Taxonomic profiles were constructed using DADA2, and statistical inferences on alpha diversity, beta diversity, and differential abundance were performed, adjusting for age, sex, smoking status, and cancer history. Sensitivity analyses additionally adjusted for cancer status, statin use, proton pump inhibitor use, and diabetes. Bacterial gene potential functions were predicted using PICRUSt2.

**Results:**

The oral microbial community composition differed in beta diversity (weighted UniFrac distance, *R* = 0.166, *p* < 0.001) and trended towards a difference in alpha diversity (Shannon index, *p* = 0.054) between patients with AAA and advanced isolated atherosclerotic disease. Ten bacterial taxa are overabundant in patients with AAA compared with those with atherosclerotic disease, most notably the genus *Streptococcus* (log2 fold change: 2.06, *p* = 0.002). PICRUSt2 predicted bacterial pathways related to protein synthesis and DNA replication that were likely to be less abundant, and pathways related to bacterial motility that were likely to be more abundant in oral cavity bacteria in subjects with AAA.

**Conclusions:**

These findings demonstrate that the oral microbiome differs between patients with AAA and those with advanced atherosclerotic disease and support further investigation of oral microbial alterations and their relationship to vascular pathologies. The known link between *Streptococcus* and cardiovascular disease makes these results particularly intriguing. Further multi-omic studies with greater power may identify bacterial species and proteins that correlate with disease presence and progression.

## Introduction

Abdominal aortic aneurysms (AAA) affect approximately 4.2% of the population (1). The disease may remain asymptomatic for years, and if it presents as a rupture, there is an estimated total mortality rate of 74% (2). Despite this, the etiology of AAA development and progression remains uncertain, and there is currently no drug therapy (3). Accumulation of inflammatory cells of all leukocyte lineages, neovascularization, and the degradation of collagen and elastin within the aortic tunica media and adventitia provide strong evidence that an inflammatory reaction plays a central role in the disease (4). Recently, upregulation of genes involved in adaptive and innate immunity in the tunica intima and media was demonstrated (5), further strengthening this claim. However, the cause of this inflammation remains unclear.

Evidence to support the involvement of microbes in the oral cavity in AAA development is building. Aneurysmal tissue has been found to contain bacteria (6–9). DNAs from various bacteria, including species known to inhabit the human oral cavity and gut, were found within aortic aneurysms (7). The role of the microbes at these locations is unclear. More recently, a bidirectional Mendelian randomization study suggested a causal relationship between the human gut microbiome and AAA formation (10). Broadly speaking, poor oral health is correlated with cardiovascular disease (11), and dysbiosis in the oropharynx may impact the development and severity of atherosclerosis, hypertension, and myocardial infarction (12, 13). Bacteria and bacterial products from this environment can enter the bloodstream and contribute to extra-oral disease processes (14). The opposite association has also been observed. Treatment for periodontal disease and improved oral hygiene mitigate systemic inflammation and reduce biomarker levels associated with atherosclerotic disease (15).

More specifically, patients with AAA tend to have more severe periodontal disease (9, 16), which provides opportunistic bacteria the ability to translocate across the oral mucosa. DNA amplification studies have identified periodontal disease pathogens, such as *Porphyromonas gingivalis,* in several vascular samples, confirming this (17). Additionally, the abundance of *P. gingivalis* in saliva samples has previously been associated with AAA diameter (16), and mechanistic studies in experimental AAA mouse models have demonstrated this bacterium can increase the AAA expansion rate (18, 19). Interestingly, randomized controlled trials using antibiotics in the treatment of AAA have been conducted (3). This was initially predicated by early evidence for the bacterium *Chlamydophila pneumonia* causing aneurysmal degeneration. In addition, the antibiotic doxycycline has been shown to reduce matrix metalloprotease activity, and a large focus was placed on this drug in the treatment of AAA. While clinical trials did not show any clinical benefit to the use of antibiotics including doxycycline (3, 20) in slowing the expansion of AAA, doxycycline did drastically reduce the population of aortic wall neutrophils and T cells by 72% and 95%, respectively (21). This is evidence that antibiotic use can significantly affect the immunological environment within the aortic wall. How this occurs is unknown, but it suggests a microbiome-mediated mechanism is plausible.

Although previous studies have linked periodontal disease (9, 16), periodontal pathogens (16–18), and salivary microbial alterations with AAA, only one study has focused on the overall oral microbial community rather than an individual pathogen (22). Because atherosclerotic disease shares many cardiovascular risk factors and inflammatory features with AAA, comparisons with healthy individuals may identify microbial signatures associated with cardiovascular disease in general rather than AAA specifically. We therefore sought to compare the oral wash microbiome of patients with AAA and those with advanced atherosclerotic disease to control for common vascular risk factors. We hypothesize that oral bacterial compositions differ between patients with AAA and those with atherosclerotic disease.

## Materials and methods

### Study cohort and sample collection

Oral wash samples were obtained from patients who enrolled in the University of Rochester, Division of Vascular Surgery's Cardiovascular Tissue Bank (STUDY00006999). This included patients with AAA and with atherosclerotic disease without AAA. Participants undergoing open aortic repair for AAA or aortoiliac occlusive disease (AIOD), endovascular aneurysm repair (EVAR) for AAA, or coronary artery bypass grafting (CABG) for CAD were enrolled. Oral wash samples were collected in the preoperative anesthesia care unit, the day of surgery, using a single-use sterile normal saline oral wash. Patients are required not to eat or drink after midnight the day prior to surgery. The samples were immediately taken to the laboratory, aliquoted, and stored at −80 °C until DNA extraction. Patients who received antibiotics within the past month were excluded.

### 16S rRNA gene amplification and sequencing

Oral wash samples were thawed, and total genomic DNA was extracted using the Zymo Quick-DNA Fecal/Soil Microbe DNA kit (Zymo Research, Irvine, CA, USA) according to the manufacturer's protocol. The V1–V3 segments of the bacterial 16S ribosomal RNA gene were amplified in collected samples using a previously studied primer set (Forward 8F: AGAGTTTGATCCTGGCTCAG, Reverse 534R: ATTACCGCGGCTGCTGG) in a 2-step process. Sample-specific barcode sequences were integrated into the forward and reverse primers in the second step. Sequencing was performed on an Illumina NovaSeq (Illumina, San Diego, CA, USA), obtaining 300 bp paired-end reads.

### Bioinformatics

Raw fastq files were demultiplexed, and taxonomic assignment was performed using DADA2 (23). Primers from all three cohorts were removed, and sequences were trimmed to begin at the same genomic location to a total read length of 260 base pairs. Given that the samples were sequenced on a NovaSeq with binned quality scores, a custom adjustment to the error rate calculation in the DADA2 pipeline was applied to enforce monotonicity and adjust the loess function's weight, span, and degree. Following this, sequence tables were merged, and chimeric sequences were removed. Taxonomy was assigned using the expanded Human Oral Microbiome Database (eHOMD) V15.22 (24), which demonstrated improved species classification compared to Silva version 138.2 (25). The DADA2 output was passed to phyloseq for the remainder of the analysis (26). Since salivary samples contain human DNA, including the mitochondrial chromosome, amplicon sequences that aligned to the family “Mitochondria” were removed.

A joint taxonomic profile was created starting at the species level. Species were retained if identified in >5% of samples for alpha diversity or >20% for beta diversity and differential abundance analyses. We performed this to reduce noise from spurious taxa arising from artifactual reads and to reduce data sparsity. Species that did not meet the thresholds were agglomerated into the genus level. The same process was completed for the genus and family levels. All sequences that were not above the threshold in sample prevalence at these taxonomic levels were merged into a single taxonomy called “unknown”. Predicted microbial functional characteristics were performed using PICRUSt2 (Phylogenetic investigation of communities by reconstruction of unobserved states) (27). PICRUSt2 was used to predict the abundance of functional gene elements in the oral community from 16S rRNA sequences. This returns a predicted abundance of KEGG Orthology (KO) gene IDs, and statistical analysis was performed as described below.

### Statistical analysis

The Shannon index was employed for alpha diversity. Linear regression was performed to test the association between disease status and both the Bacteroidetes-to-Firmicutes ratio and the Shannon index. The weighted UniFrac distance was used for beta diversity (28). Principal component analysis was performed on the beta diversity, and statistical inference was conducted using permutational multivariate analysis of variance (PERMANOVA) (29). Differential abundance analysis was performed using LinDA, a regression-based method that accounts for compositional effects, resulting in a lower false-positive rate than other methods (30). The covariates age, sex, smoking status, and cancer history were used in all the analyses above, given their established associations with oral microbiome variation. Additional sensitivity analyses were performed, removing subjects with cancer and adjusting for statin use, proton pump inhibitor use, and diabetes. Moreover, differential abundance between coronary artery disease and aortoiliac occlusive disease in the control cohort was assessed. Correlation between patient laboratory values and each of the differentially abundant taxa identified above was assessed using Spearman's rank-order correlation on center log ratio (CLR)-transformed values. Given the exploratory nature of this laboratory correlation analysis, no correction for multiple testing was applied. LinDA was again used with adjustments for age, sex, and smoking status for the analysis of differentially abundant KO gene IDs that were predicted using PICRUSt2. Gene set enrichment analysis of all KO gene IDs was performed using ggpicrust2 (31).

## Results

A total of 73 oral wash samples were collected from 45 subjects with AAA and 28 with atherosclerotic disease. Patients with AAA were, on average, older and more often male, with a higher rate of concomitant cancers. In addition, for patients with relevant clinical laboratory values within six months of surgery, patients with AAA had lower average platelet counts and lower average triglycerides (Table 1).

**Table 1:** Basic cohort characteristics.

Characteristic	Cohort		*p*
	AAA	Atherosclerotic Control	
*n*	45	28	
Age (mean (SD))	73.04 (9.16)	62.68 (11.00)	<0.001
Sex (% Male)	38 (84.4)	19 (67.9)	0.169
BMI (mean (SD))	28.40 (7.74)	27.21 (6.42)	0.503
Diabetes (%)	11 (25.0)	10 (35.7)	0.478
Cancer (%)	13 (28.9)	1 (3.6)	0.021
Autoimmune Disease (%)	5 (11.1)	4 (14.3)	0.972
Statin Use (%)	38 (88.4)	23 (82.1)	0.698
Proton Pump Inhibitor Use (%)	15 (34.9)	10 (37.0)	1.000
Self-Reported Dental Procedure (%)	7 (20.0)	4 (26.7)	0.882
Current Smoker (%)	19 (42.2)	8 (28.6)	0.355
AAA Diameter (mean (SD))	5.80 (1.35)	2.38 (1.02)	<0.001
Platelet Count (1000s) (mean (SD))	209.93 (67.38)	251.61 (89.52)	0.039
Lymphocyte Count (mean (SD))	1.74 (0.94)	1.96 (0.68)	0.346
Monocyte Count (mean (SD))	1.04 (1.47)	0.67 (0.28)	0.241
HbA1C (mean (SD))	5.99 (0.94)	6.44 (1.41)	0.175
Total Cholesterol (mean (SD))	142.49 (42.98)	144.88 (35.87)	0.861
Triglycerides (mean (SD))	114.05 (56.68)	176.88 (97.69)	0.024
HDL (mean (SD))	45.89 (10.84)	41.12 (15.38)	0.291
LDL (mean (SD))	80.00 (31.99)	79.50 (37.55)	0.958

### Diversity

**Figure 1:**
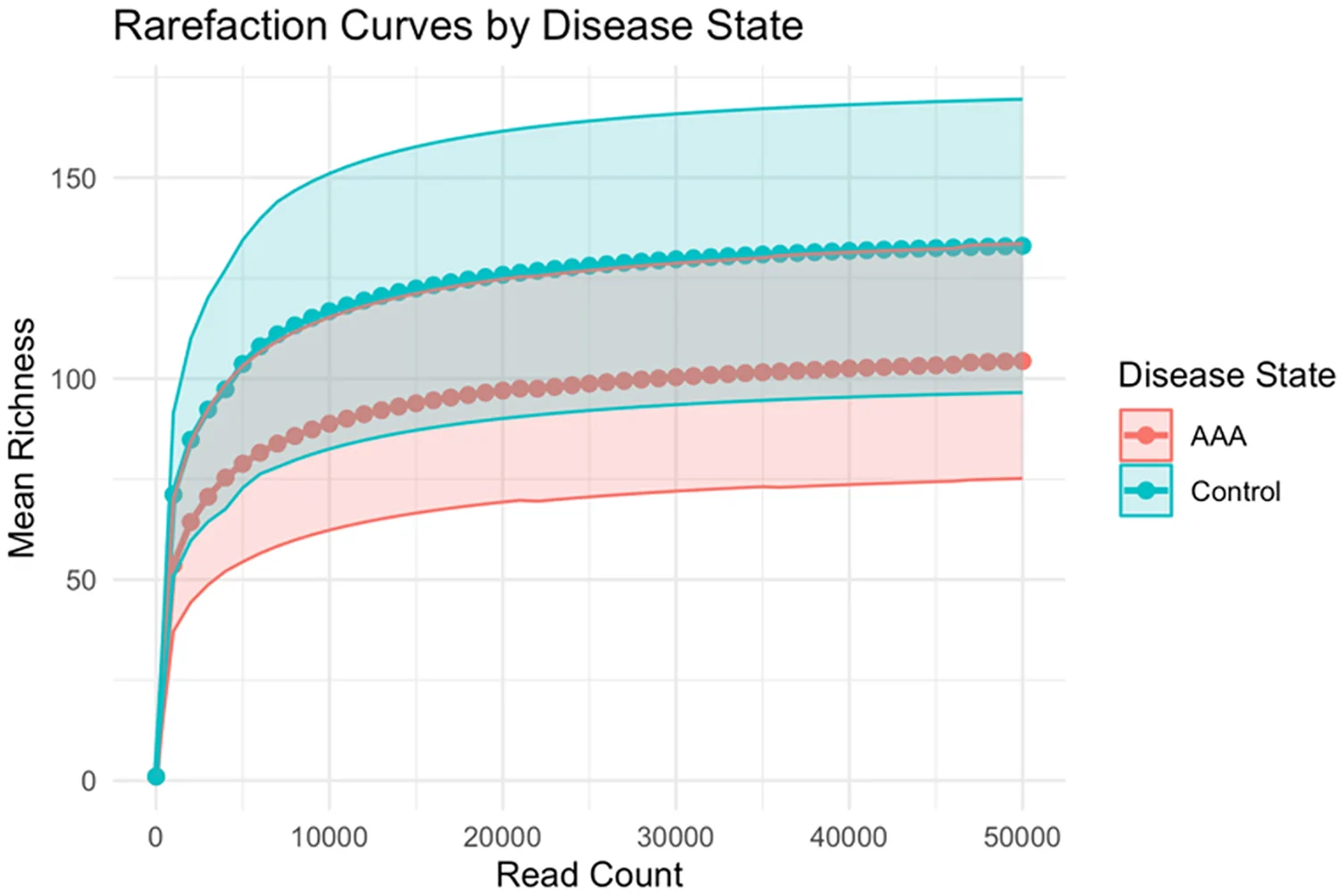
Rarefaction curves for oral wash samples from patients with AAA and from patients with atherosclerosis. The curves demonstrate saturation of taxonomies at the obtained sequencing depth. They also demonstrate a lower mean richness in AAA oral wash samples than in atherosclerotic control samples.

**Figure 2:**
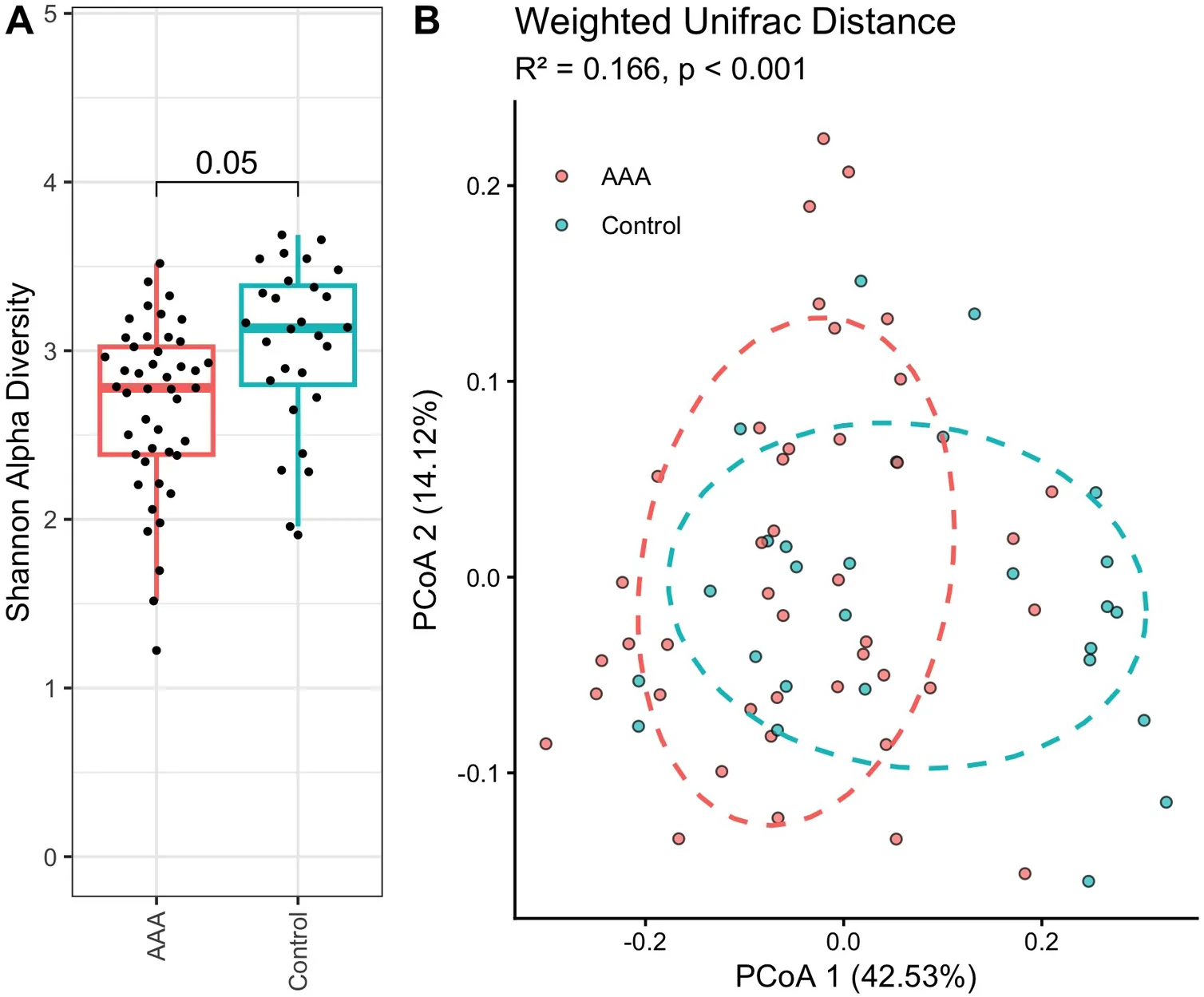
Alpha and Beta diversity measures capture differences in oral wash bacterial communities by disease state. **(A)** On average, the Shannon alpha diversity measure trended towards lower in oral wash samples from subjects with AAA (2.67 ± 0.51) compared to those with atherosclerotic disease (3.03 ± 0.50, *p* = 0.054). **(B)** Beta diversity analysis using weighted UniFrac distance identified 16.6% of the variation in taxa composition between samples could be explained by the disease state (*p* < 0.001).

**Figure 3:**
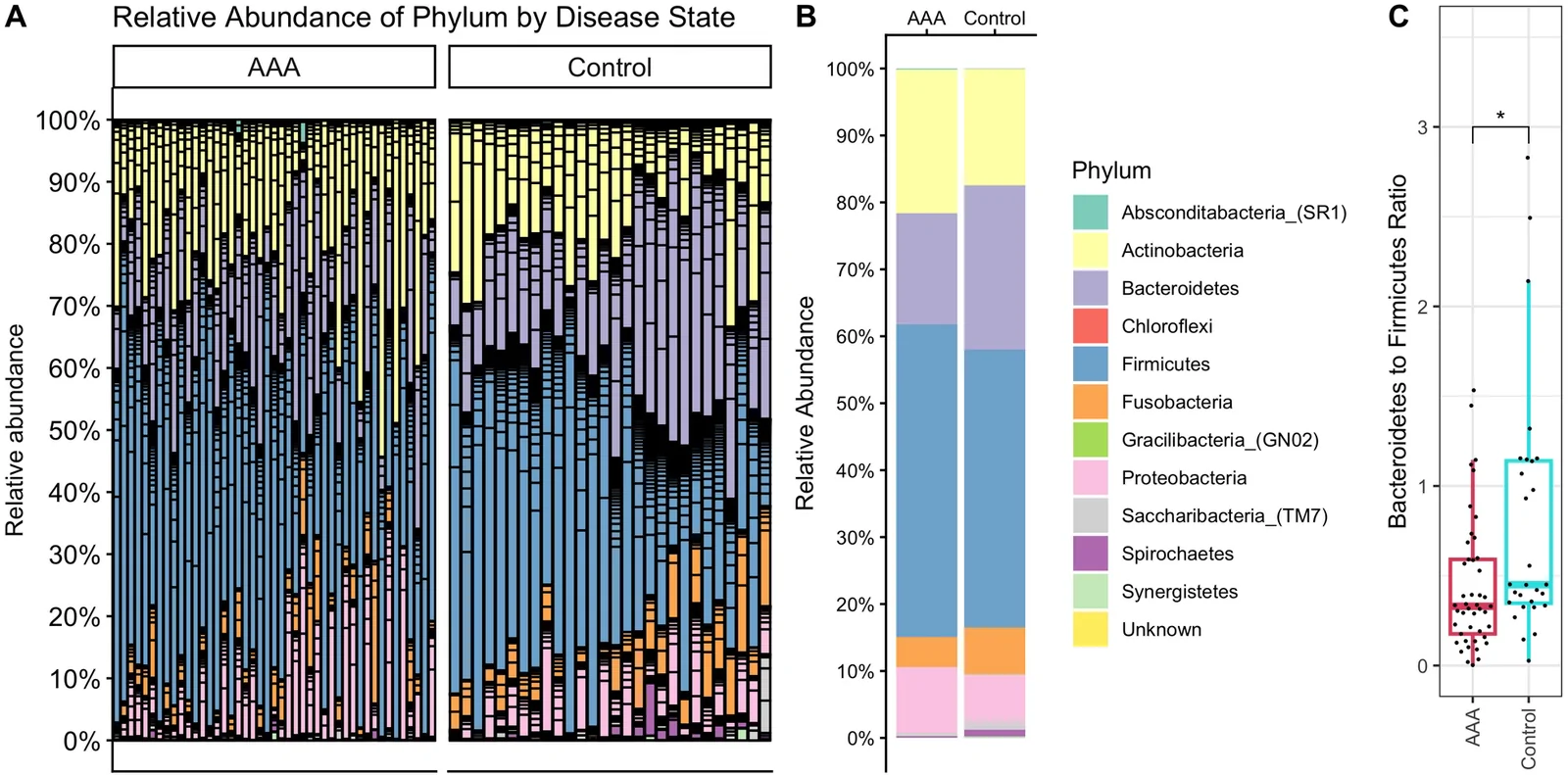
Relative abundance of taxa by phylum. **(A)** Bar plot of the relative abundance of each phylum within each sample, grouped by disease state. **(B)** The relative abundance of each phylum in all samples by disease state. **(C)** Plot of the Bacteroidetes-to-Firmicutes ratio in AAA samples (0.44 ± 0.37) relative to control samples (0.79 ± 0.71, *p* = 0.037).

For alpha diversity, a total of 377 taxa were included after filtering with 230 unique species, 130 unique genera, and 62 unique families. The rarefaction curve, derived by plotting the average number of observed taxa per sample against the subsampled number of reads, demonstrates that richness in both groups approaches saturation at the obtained sequencing depth. Therefore, sequencing was sufficiently deep to cover the majority of bacteria within the oral wash community. In addition, the rarefaction curves showed that the taxonomic richness in AAA oral wash samples was lower than that in atherosclerotic disease samples (Figure 1). A similar result was seen for alpha diversity, as measured by the Shannon index, which trended towards lower in oral wash samples from AAA (2.67 ± 0.51) patients than in atherosclerotic disease controls (3.03 ± 0.50, *p* = 0.054, Figure 2A). The relative abundance at the phylum level by disease in each individual sample is shown in Figure 3A, and the relative abundance of each phylum over all samples in a disease state is shown in Figure 3B. There was also a decrease in the Bacteroidetes-to-Firmicutes ratio in AAA samples (0.44 ± 0.37) relative to control samples (0.79 ± 0.71, *p* = 0.037, Figure 3C).

For the beta diversity analysis, 171 taxa were retained after filtering, including 79 unique species, 83 unique genera, and 47 unique families. After controlling for age, sex, and smoking status, PERMANOVA identified a difference in bacterial composition between disease states (*R*^2^ = 0.166, *p* < 0.001, Figure 2B).

### Differential abundance

**Figure 4:**
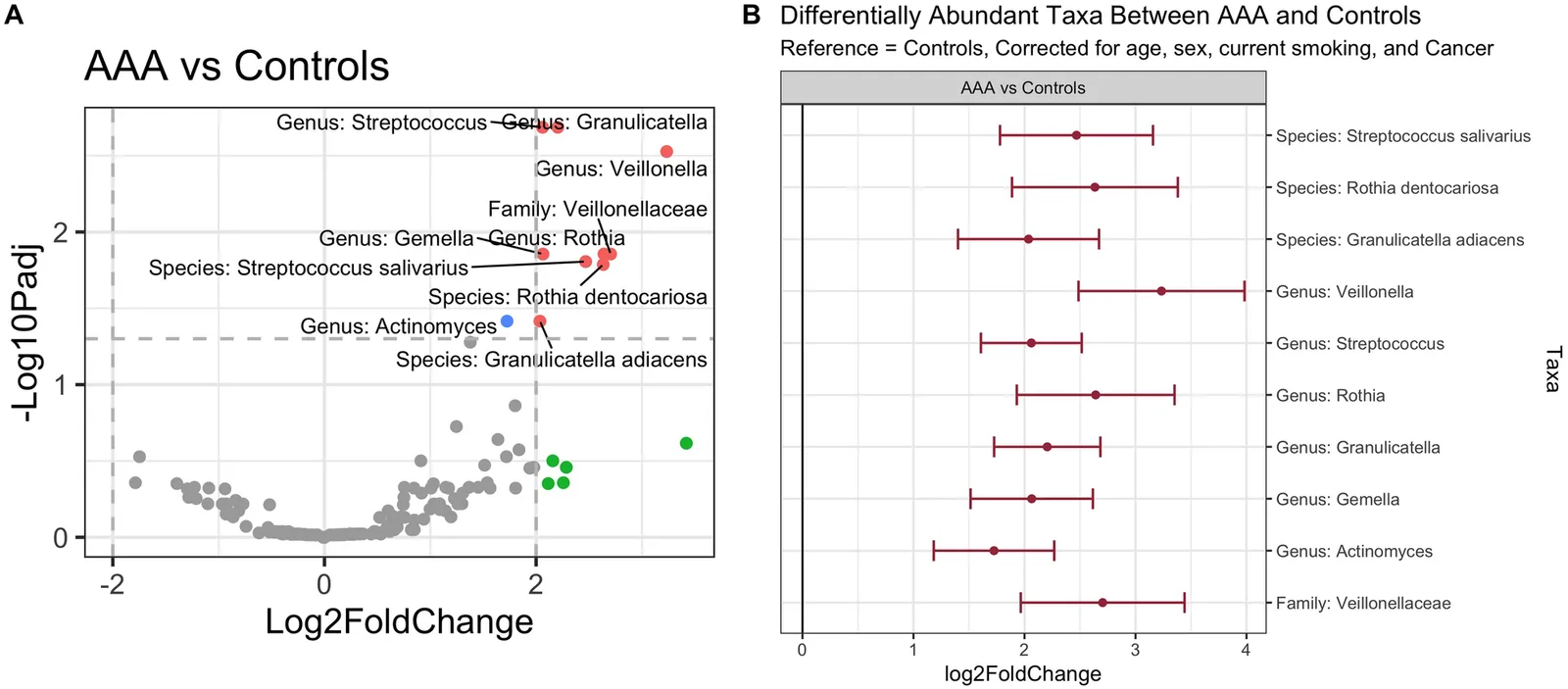
Taxonomic differential abundance by disease state. **(A)** Volcano plot of differentially abundant bacterial species, genera, and families between patients with AAA and atherosclerotic controls using LinDA with the control cohort as the reference. **(B)** Forest plot of the differentially abundant bacterial taxa with the log2 fold change between AAA and the atherosclerosis control cohort plotted. All taxa are more abundant in the AAA cohort than in the atherosclerosis cohort.

After adjustment for multiple testing, there were 10 differentially abundant taxa between the AAA and control cohorts, all with higher abundance in the AAA cohort. This set of 10 included 3 at the species level, 6 at the genus level, and 1 at the family level (Figure 4).

**Figure 5:**
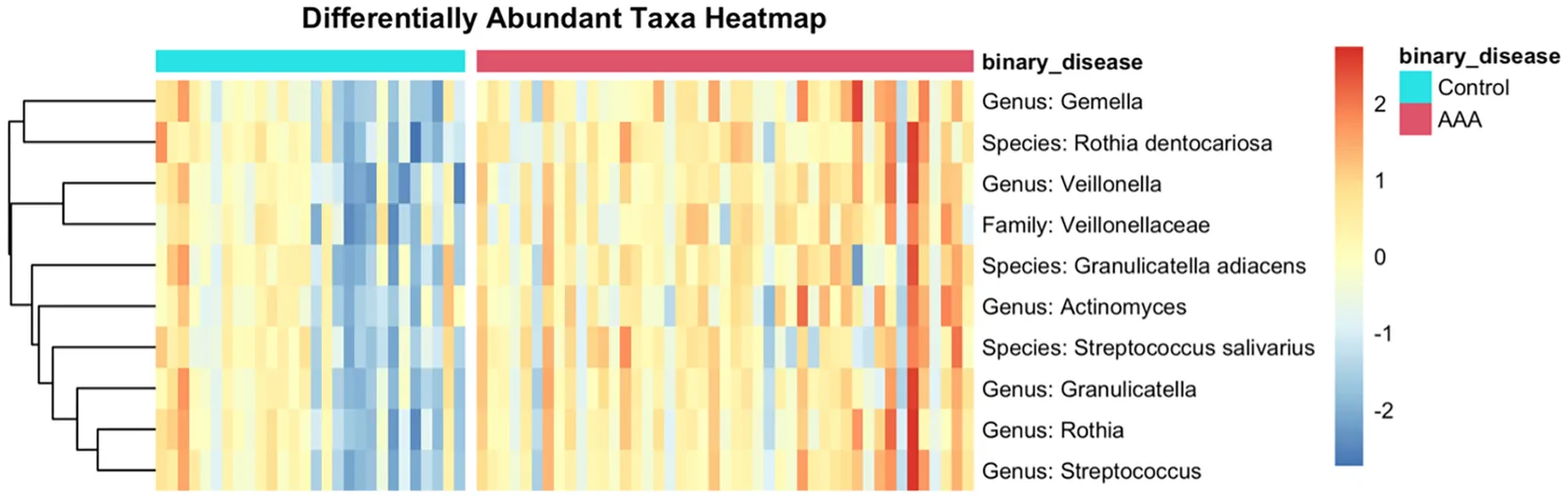
Heat map of differentially abundant taxa. The centered log-ratio transformation for each taxon within each sample is plotted, and samples are grouped by disease state.

The most abundant species in AAA of the differentially abundant tax was *Streptococcus salivarius*, with a median relative abundance of 2.5% (IQR:1.2%–5.4%) in the AAA cohort and 1.8% (IQR:0.7%–4.5%) in the control cohort. *Streptococcus salivarius* demonstrated a 2.47 log2-fold increase in AAA when compared to controls on linear differential abundance analysis after correcting for age, sex, smoking status, and cancer diagnosis (p_adjusted_ = 0.016). The species *Granulicatella adiacens* and *Rothia dentocariosa* were also differentially abundant (Figure 4; Supplementary Table 1). The most common differentially abundant genus was *Streptococcus* with a median abundance of 26% (IQR: 19.2%–35.1%) in the AAA cohort and 22% (IQR:11.2%–31.2%) in the control cohort. The *Streptococcus* genus demonstrated a 2.06 log2-fold increase in AAA when compared to atherosclerotic disease on linear differential abundance analysis after correcting for age, sex, smoking status, and cancer diagnosis (p_adjusted_ = 0.002). Additional differentially abundant genera included *Actinomyces, Gemella, Granulicatella, Rothia,* and *Veillonella* (Supplementary Tables 1, 2). The family *Veillonellaceae* was also more abundant in the AAA cohort compared to controls (Figure 4 and Supplementary Table 1). Further details on the differentially abundant taxa are provided in Supplementary Table 1. A heatmap depicting the relative abundance of these 10 differentially abundant taxa in each sample, grouped by disease state, is shown in Figure 5. To gain an appreciation of which bacterial species may be driving the genus-level association, Supplementary Table 2 provides LinDA results for all *Streptococcus*, *Granulicatella*, and *Veillonella* species. The primary contribution to the *Streptococcus* genus appears to be *Streptococcus salivarius* with a log2fold change of 2.47 (p_adjusted_ = 0.016). *Streptococcus mitis* may also be contributing to the association with a log2fold change of 2.26 however, the result was not significant after correction for multiple testing (p_adjusted_ = 0.439, Supplementary Table 2). Given the prior association of AAA with periodontitis, the results of the LinDA analysis for common periodontal pathogens are reported in Supplementary Table 3. The Genera *Prevotella*, *Porphyromonas*, *Fusobacterium*, *Tannerella*, *Aggregatibacter*, and *Treponema* are listed, and, interestingly, no positive association among these genera or species was identified.

To assess the robustness of the primary findings, several sensitivity analyses were performed (Supplementary Figure 1). First, all patients with a history of malignancy were excluded, and differential abundance analysis was repeated on the remaining 59 subjects. The genera *Granulicatella*, *Streptococcus*, and *Veillonella* remained significantly more abundant in patients with AAA compared with those with atherosclerotic disease (Supplementary Figure 1A). Second, the differential abundance analysis was repeated using an expanded multivariable model on all subjects that adjusted for diabetes, statin use, proton pump inhibitor use, in addition to age, sex, smoking status, and cancer status. The genera *Granulicatella*, *Streptococcus*, and *Veillonella* again remained significantly enriched in the AAA cohort (Supplementary Figure 1B). Third, because triglyceride measurements were unavailable for all subjects, differential abundance analysis was repeated in the subset of patients with triglyceride levels measured within 6 months of oral sample collection. In this restricted analysis of 35 samples, *Gemella sanguinis* remained significantly more abundant in the AAA cohort (Supplementary Figure 1C). Finally, to evaluate potential heterogeneity within the atherosclerotic disease control cohort, differential abundance analysis was performed comparing patients with aortoiliac occlusive disease and coronary artery disease. Five taxa were identified as differentially abundant between these two control subgroups. Of these, only *Rothia dentocariosa* was also differentially abundant in the primary comparison between AAA and the combined atherosclerotic disease cohort (Supplementary Figure 1D).

### Correlation of differentially abundant species with laboratory values

**Figure 6:**
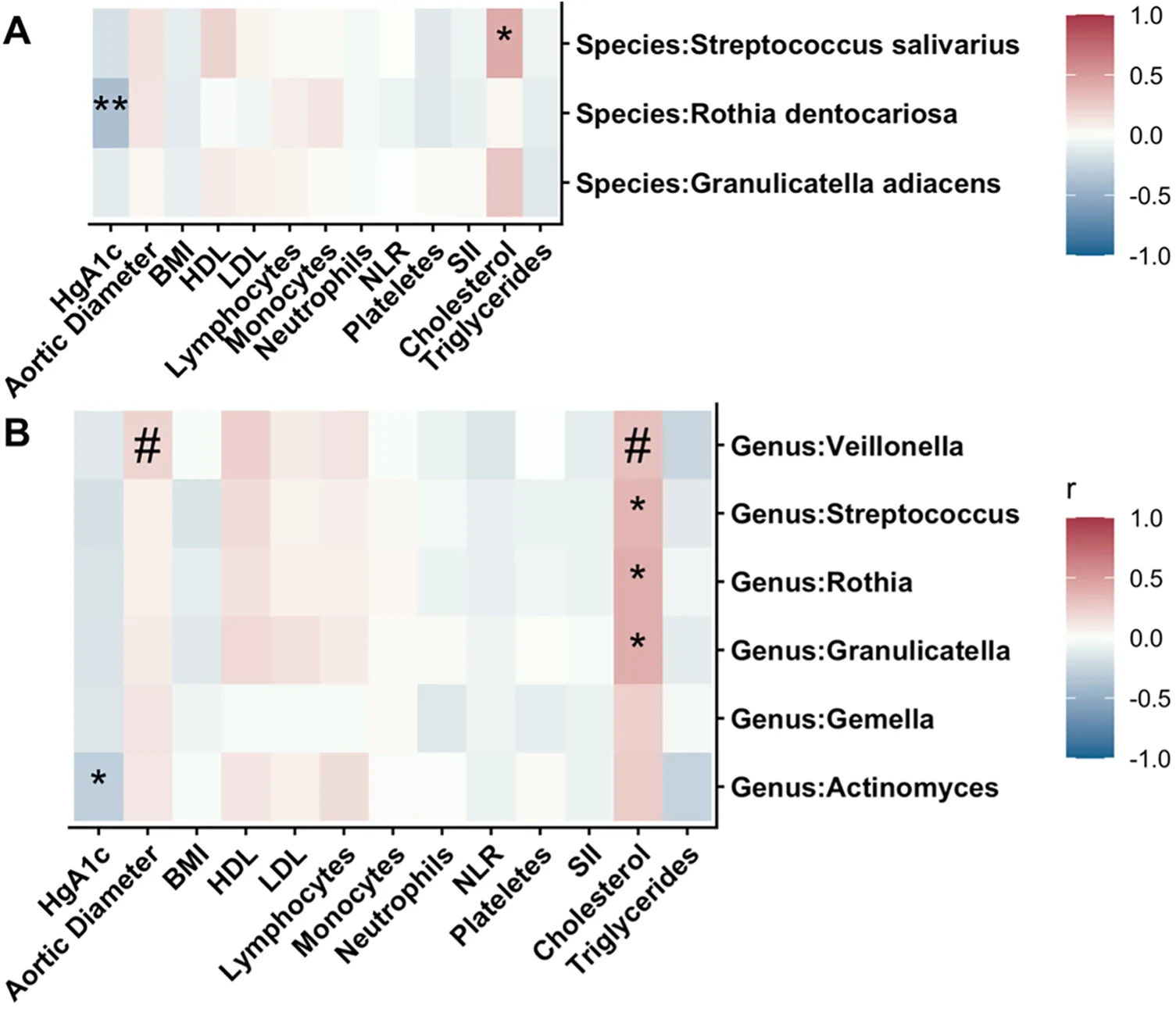
Correlation of patient laboratory values to differentially abundant Species and genera. Heatmap displaying Spearman's rank-order correlation coefficient between the **(A)** species and the **(B)** genus centered log ratio and various laboratory values, obtained within the last 6 months, for differentially abundant taxa. # = *p* < 0.1, * = *p* < 0.05, ** = *p* < 0.01. There are 4 taxa positively associated with cholesterol levels. This data is not corrected for multiple testing. No taxa were significantly associated with aortic diameter.

Species identified as differentially abundant between AAA and the control cohort were assessed for their correlation to laboratory values using Spearman's rank-order correlation. Four taxa demonstrated a potential positive correlation with patients’ cholesterol levels. *Streptococcus salivarius*, *Streptococcus, Rothia*, and *Granulicatella* were all positively correlated with cholesterol with R of 0.25, 0.35, 0.28, and 0.23, respectively (Figure 6). *Rothia dentocariosa* was negatively correlated with patients’ hemoglobin A1c levels. Finally, no association between these taxa and aortic diameter was identified. It must be noted that this was an exploratory analysis, and no correction for multiple testing was performed.

### Prediction of bacterial functional states using PICRUSt2

**Figure 7:**
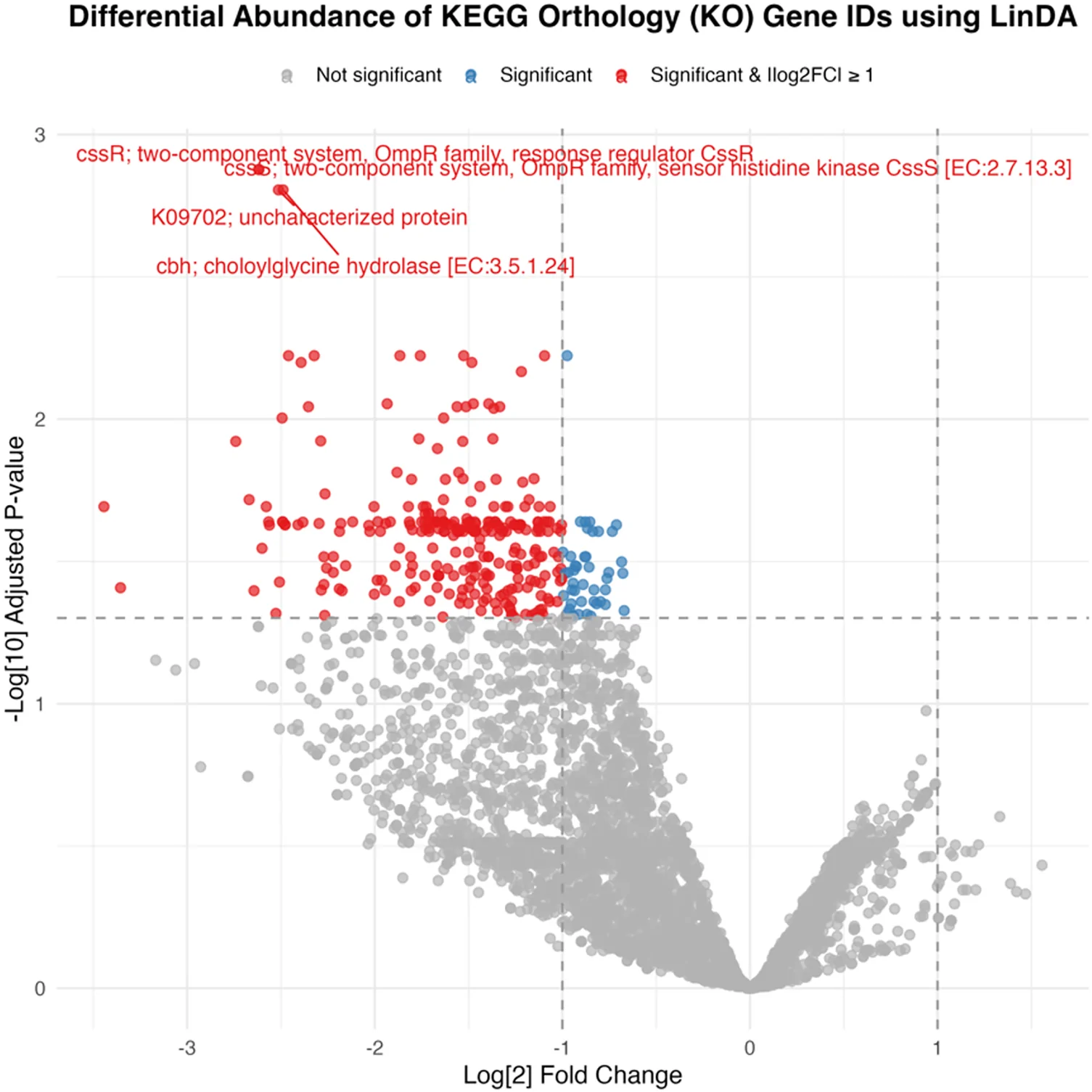
Predicted differential abundance of KEGG orthology gene IDs by disease state. Volcano plot of differentially abundant KEGG Orthology gene IDs between patients with AAA and atherosclerotic controls using linear differential abundance (LinDA) analysis with correction for age, sex and smoking status.

**Figure 8:**
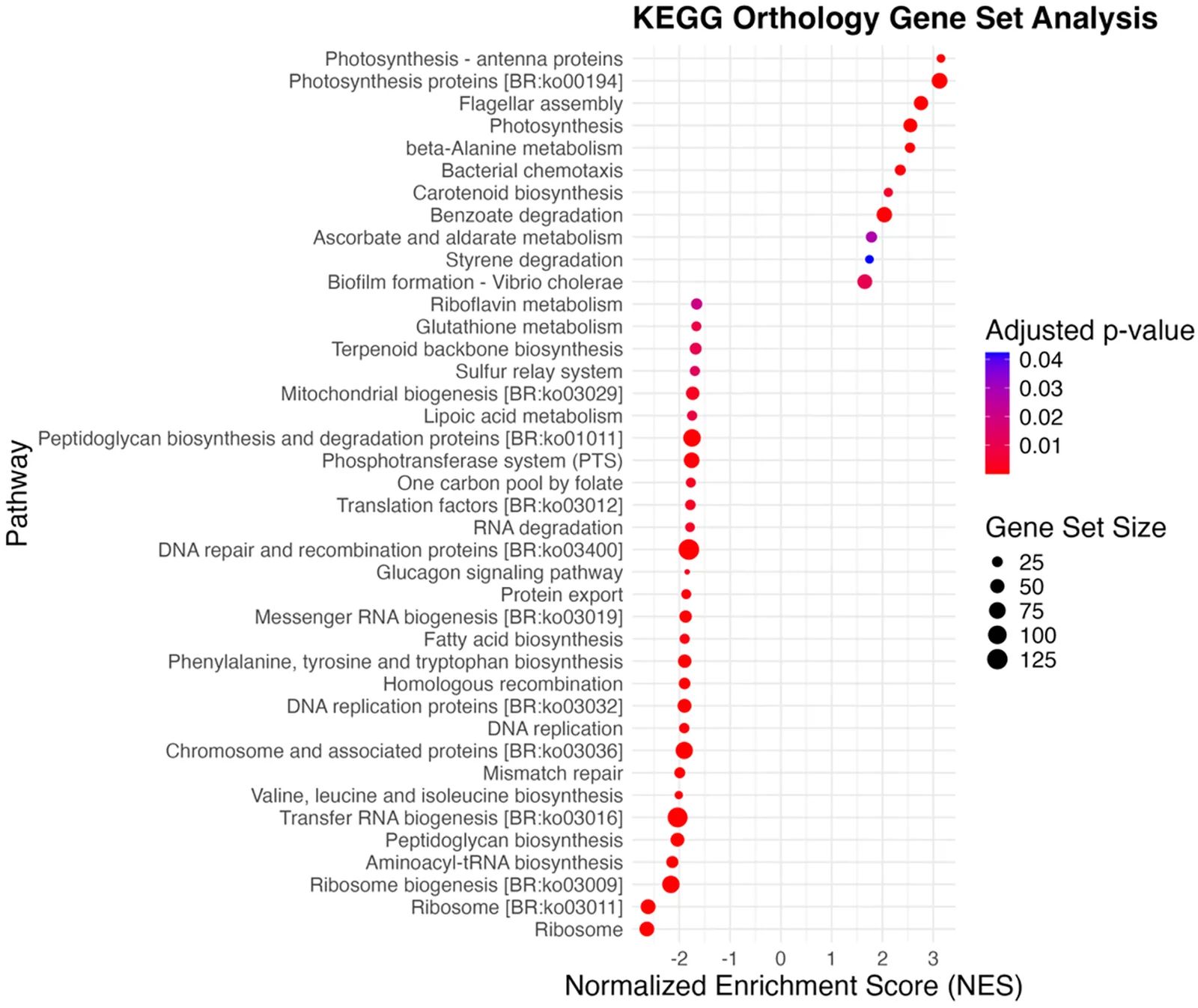
Predicted KEGG orthology gene set analysis by disease state. Forest plot of the top 40 differentially abundant KEGG orthology gene sets between AAA and the control cohort, ordered by normalized enrichment score.

PICRUSt2 predicted 314 differentially abundant gene IDs between oral wash communities in patients with AAA and those with atherosclerotic disease. Notably, these were all less abundant in AAA with cssS (Log2 Fold Change: −2.62, adjusted *p* = 0.0012) and cssR (Log2 Fold Change: −2.62, adjusted *p* = 0.0012), both components of the two-component stress response system, predicted to be the most differentially abundant (Figure 7; Supplementary Table 4). Given that 314 gene IDs were less abundant in AAA, a gene set analysis was performed (31). Several pathways related to metabolic activity, including ribosome biogenesis (Normalized Enrichment Score (NES): −2.17, adjusted *p* < 0.001), transfer RNA biogenesis (NES: −2.03, adjusted *p* < 0.001), fatty acid biosynthesis (NES: −1.89, adjusted *p* < 0.001), DNA mismatch repair (NES: −1.99, adjusted *p* < 0.001), and DNA repair (NES: −1.81, adjusted *p* < 0.001), were predicted to be downregulated in subjects with AAA. Conversely, pathways related to bacterial motion, such as flagellar assembly (NES: 2.76, adjusted *p* < 0.001) and bacterial chemotaxis (NES: 2.35, adjusted *p* < 0.001), were predicted to be increased in the oral wash bacterial community of subjects with AAA (Figure 8; Supplementary Table 5).

## Discussion

The above analysis of 16S rRNA sequencing data from human oral wash samples from subjects with AAA and those with atherosclerotic disease demonstrates differences in these communities between the two disease states. In addition, after adjusting for age, sex, smoking status, and cancer, 10 bacterial taxa were identified as more abundant in the oral cavity of patients with AAA compared to those with atherosclerotic disease. Interestingly, despite the established association between AAA and periodontal disease, classical periodontal pathogens were not differentially abundant in our cohort, suggesting that the observed microbial differences extend beyond the canonical periodontal pathogen signature described previously.

This work extends previous investigations linking oral health and AAA by demonstrating that differences in the oral microbial community persist even when patients with AAA are compared with controls with advanced atherosclerotic disease rather than with healthy subjects. This experiment's design may better isolate microbial signatures associated with aneurysmal disease rather than cardiovascular disease more broadly since both cohorts share many traditional cardiovascular risk factors. The present study should be interpreted within the context of the growing body of literature linking oral health and AAA. Previous clinical studies have demonstrated that patients with AAA have a higher prevalence of periodontitis, greater periodontal disease severity, and increased detection of periodontal pathogens such as *Porphyromonas gingivalis* in oral and aneurysmal tissues (9, 12, 16, 18). Experimental studies have further supported the biological plausibility of this association by demonstrating that oral pathogens can accelerate aneurysm progression in animal models (18, 19). These findings extend the literature by demonstrating that differences in the oral microbial community persist between patients with AAA and those with advanced atherosclerotic disease, beyond the classic periodontitis pathogens. Importantly, clinical markers of periodontal severity, such as periodontal probing depth, clinical attachment loss, bleeding on probing, dentition status, were not captured in this study. Consequently, we cannot determine whether the observed microbial differences reflect AAA-associated microbial alterations, differences in periodontal disease burden, or both.

The identification of *Streptococcus* and the related Genera of *Gemella*, and *Granulicatella* is particularly interesting since recent work exploring the differential abundance of oral bacteria between healthy controls and AAA patients identified several differentially abundant genera, including *Streptococcus*, *Gemella, and Granulicatella* (22). Furthermore, a recent metagenomics study of stool samples identified *Streptococcus* species and other oral taxa as differentially abundant in the lower GI tract of subjects with AAA compared to controls (32). The validation of this bacterial genus across multiple datasets provides promising support for the association between AAA and the differential abundance of oral *Streptococcus*. The previously reported work (22) identified several other taxa as differentially abundant; however, the methods used in that study, LEfSe (Linear Discriminant Analysis Effect Size), have higher false-positive rates because they do not account for the compositional nature of microbiome data and do not correct for bias, compared with the methods used here (33). LinDA explicitly accounts for the compositional nature of microbiome sequencing data while controlling the false discovery rate, thereby reducing the likelihood of spurious differential abundance findings. Consequently, our results complement previous studies by confirming several shared microbial associations using a statistical framework specifically developed for compositional microbiome data.

Although the unadjusted absolute differences in median relative abundance were modest for some taxa, these descriptive values do not account for differences in age, sex, smoking status, cancer history, diabetes, or medication use. After the nature of compositional data is considered and covariates are adjusted for in the differential abundance models, several taxa showed log2 fold changes >2. This suggests the observed differences may be biologically meaningful despite relatively small unadjusted differences in median abundance. These findings are best interpreted as evidence of a community-level microbial shift associated with AAA rather than the expansion of a single dominant pathogen, and they should be validated in future studies.

Comparing the oral microbiome in those with AAA to that in those with atherosclerotic disease offers a novel insight into the role of the oral microbiome in the development of aortic aneurysmal pathology. Historically, degenerative aneurysmal disease has been considered to be the result of atherosclerotic damage to the integrity of the vessel wall. Although atherosclerotic disease frequently coexists with AAA, it is unlikely to be the primary driver of this immune phenotype. Histologically, the inflammation in aortic atherosclerosis is largely confined to the inner media and intima around the necrotic core. The media is relatively preserved, and lymphoid follicles are rarely present. In contrast, AAA is marked by destruction of both intima and media with elastin degradation and collagen deposition. Diffuse transmural inflammation is present with prominent adventitial lymphoid aggregates (34). Additionally, most atherosclerotic lesions in AAA are minimal compared with AIOD (34). These are distinct histopathological processes that share common risk factors such as smoking, hypertension, and dyslipidemia (35). Consistent with this distinction, our atherosclerotic control cohort had significantly higher triglyceride levels, despite similar HDL and LDL values. The findings reported here demonstrate that there is a difference in the oral wash microbiome between these two entities despite shared vascular risk factors. Albeit, triglyceride levels remained difficult to account for, given the limited number of preoperative lab values obtained.

Plausible mechanisms by which oral bacteria can contribute to AAA pathogenesis include 1) direct bacterial seeding of the arterial wall, leading to the establishment of a chronic inflammatory state, or 2) a chronic immune modulation within the oral cavity, resulting in a low-grade autoimmune response to arterial wall components. First, direct exposure of oral bacteria to the aortic wall via hematogenous spread is a common occurrence. Recurrent and transient bacteremia arising from the oral cavity during procedures as common as toothbrushing is a described phenomenon, providing repeated opportunities for vascular exposure to circulating microbes (36). Consistent with this, bacterial DNA from species primarily found in the oral cavity has been identified in aortic aneurysms (7). Nutritionally variant streptococci (NVS), members of the viridans group streptococcus–like organisms that commonly inhabit the oral cavity, are well-recognized causes of bacteremia and septicemia and account for an estimated 4%–8% of all cases of infective endocarditis (37). Seeding the aortic wall with bacteria may initiate a local inflammatory response. The work presented here identified an overabundance of several Gram-positive bacteria that express lipoteichoic acid as a structural component of their cell wall. This can be a potent activator of innate immune responses. The molecule can promote macrophage activation, elastin degradation, and vascular smooth muscle cell apoptosis—processes central to aortic wall weakening and aneurysmal degeneration (38). Additionally, these bacteria can potently induce pro-inflammatory cytokines that drive GM-CSF production and MMP release, another mechanism core to AAA pathology (39, 40).

A mechanism of direct arterial exposure is further supported by the results from the predicted functional elements by PICRUSt2. When genes were grouped into pathways, pathways downregulated in AAA patients were those implicated in growth, replication, and repair, including ribosome production, fatty acid synthesis, DNA repair, and mismatch repair. However, in the AAA cohort, genes involved in motility and environmental navigation, such as those for flagellar assembly and chemotaxis, were upregulated. Previous studies have demonstrated similar findings: disease-associated microbial communities showed reduced metabolic pathways, with a concomitant increase in pathways associated with motility and environmental navigation. A prior analysis of oral bacteria in recurrent aphthous stomatitis (RAS) using PICRUSt2 showed proportionally lower predicted metabolic functions in bacterial colonies and higher functional categories related to cell motility and environmental adaptation in patients with RAS (41). Such a shift in predicted functional pathways is consistent with microecological adaptation strategies, in which microbial communities adapt to an altered host environment, for example, following cigarette smoke exposure. These analyses predicted overrepresentation of bacterial motility-associated pathways in the oral cavity, specifically an overabundance of flagellar and chemotaxis proteins in AAA patients. This raises the possibility that microbial communities with greater predicted motility may have an increased capacity for host interaction and transient bacteremia, although this cannot be inferred directly from PICRUSt2 predictions. In addition, flagellin is a highly immunogenic peptide that activates both the innate and adaptive immune systems. It is a potent activator of toll-like receptor (TLR) 5 and can induce an adventitial-initiated perivasculitis, a pattern similar to human AAA (42). Furthermore, this pathway can cause vascular smooth muscle cell migration (43). Thus, oral dysbiosis in patients with abdominal aortic aneurysms (AAA) may contribute to chronic vascular inflammation and progressive aortic wall degeneration.

Second, it is widely accepted that the human microbiome is required to support proper immune system training. The human immune system adapts over time with continuous interaction with commensal microbes. Therefore, changes in microbial community composition or expression can shift host immunity towards tolerance or activation. Specifically, infections with these commensal bacteria, such as Streptococci, are well known to cause delayed cardiovascular disease through immune-mediated mechanisms. The most well-known of these is rheumatic heart disease (RHD). More recently, both oral and gut microbiome composition have been shown to influence the development and severity of RHD (44), further supporting the concept that human microbial communities can prime maladaptive immune responses. Given this, the differential abundance of commensal bacteria identified in this work between AAA and atherosclerotic patients may represent a fundamental difference in host immune phenotype. Aneurysmal disease is increasingly recognized as a chronic inflammatory disease, yet the origin remains unknown. Changes in the host microbiome composition and antigenic expression could explain the source of inflammation. Future work should focus on elucidating the inciting antigens.

Importantly, the results in this study show an association between specific bacteria and AAA when compared to an atherosclerosis control cohort. The question of causality remains unresolved in this cross-sectional study, despite the well-understood mechanisms of bacterial translocation and subsequent inflammation described above. Specifically, the functional pathways identified were inferred computationally from 16S rRNA profiles rather than measured directly by metagenomic or metatranscriptomic sequencing; therefore, these observations should be considered hypothesis-generating as this is a predictive tool. However, the current literature supports an association between oral dysbiosis and abdominal aortic aneurysm and provides biologically plausible mechanisms worthy of further investigation.

This work has several strengths. First, the statistical methods are robust. Statistical approaches to address compositional effects were employed to reduce the false discovery rate (30). This has not been used in prior reports. In addition, an attempt was made to control for confounding factors, including patient age, sex, smoking history, and cancer status via statistical methods, dietary habits via timing of sample collection, and antibiotic used by removal of antibiotic exposure. Multiple sensitivity analyses demonstrated that the primary findings were robust to alternative model specifications and cohort restrictions, including exclusion of patients with malignancy, adjustment for additional clinical covariates (diabetes, statin use, and proton pump inhibitor use), restriction to subjects with available triglyceride measurements, and assessment of heterogeneity within the atherosclerotic disease control cohort. Although the smaller triglyceride-restricted analysis reduced the number of statistically significant taxa, this was largely due to the smaller sample size, and the overall direction of the observed microbial differences remained consistent, supporting the robustness of the primary findings.

Patient samples were obtained on the morning of their operative day, after they had fasted and had similar diurnal hormonal profiles. However, a patient's diet will affect their oral microbiome in ways that could not be fully characterized or controlled in this study. The overnight fasting state of these patients may help mitigate this discrepancy. The assessment of smoking history is subject to recall bias and is often poorly captured during patient interviews. As such, the implication of active, passive, or a history of smoking may not be well-characterized and distributed amongst our patient cohorts. However, no significant statistical variation in smoking exposure between the two cohorts was identified.

This work is limited to 16S ribosomal RNA amplicon sequencing data. While this technique can identify taxonomic differences between samples, it is less precise than metagenomic shotgun sequencing and cannot comment on bacterial functions that may be related to the disease process. Using PICRUSt2 to predict functional elements of the microbiome community can be inaccurate. Having a specific map of the functional components of the microbiome can be impactful. This is particularly important given the vast differences in functions between species within a genus. For example, species within the genus *Streptococcus* are known to have very different functions. *Streptococcus mutans* has a more aggressive phenotype and contributes significantly to dental caries. Conversely, *Streptococcus parasanguinis* is an important commensal bacterium that can establish a healthy biofilm. Given this, aggregating species at the genus level and, furthermore, at the family level can be misleading. Therefore, there is a limitation to any association between a taxonomic differential abundance at the genus level and higher with disease states. Further work should leverage existing multi-omic technologies such as metagenomics, metabolomics, and proteomics to gain a better understanding of bacterial functional differences between patients with AAA and controls. Another major limitation is the absence of detailed oral health phenotyping and clinical markers of periodontal severity, as discussed previously. Although sensitivity analyses demonstrated minimal influence of disease subtype within the control cohort, combining patients with AIOD and CAD may still obscure disease-specific microbial differences that larger future studies should evaluate. Finally, given the exploratory nature of the study and the lack of a standard analytical power calculation for LinDA, we did not perform one. At the current sample size of 45 AAA and 28 controls, this study is likely best powered to detect moderate-to-large taxon-level differences and is underpowered to detect smaller differences after FDR correction. To confirm these findings, given the small sample size and additional limitations discussed above, external validation in an independent, geographically distinct cohort should be conducted. However, it is interesting that *Streptococcus* was also identified as differentially abundant between AAA and the healthy control group in a previous work from China (22). This provides additional validation to the presented work here.

Despite the limitations of this study, the work provides evidence of an association between oral microbial communities in patients with AAA and those with advanced atherosclerotic disease, independent of classic periodontal pathogens. These observed differences remain robust across multiple sensitivity analyses. Although this cross-sectional study cannot establish causality, the consistent enrichment of *Streptococcus* and related taxa provides a strong rationale for future mechanistic investigations. Further work will determine the temporal variation in the oral microbiome and its relationship to AAA progression and will further characterize bacterial functional states that are differentially abundant in AAA. This future work may identify microbial mechanisms contributing to vascular inflammation and provide targets for future translational investigation.

## Conclusion

The data here demonstrate that oral bacterial communities differ between patients with AAA and those with atherosclerosis without AAA. Several oral microbiota taxa, specifically *Streptococcus* and related Gram-positive taxa, are differentially abundant between these two cohorts, which is robust to sensitivity analyses. Predicted functional profiling further suggests a shift toward motility- and chemotaxis-associated pathways, with relative reductions in metabolic and replicative functions. These findings support an association between oral dysbiosis and aneurysmal pathobiology when compared to patients with advanced atherosclerotic disease. Future longitudinal and mechanistic studies incorporating metagenomics, metatranscriptomics, and host immune profiling will be essential to determine whether these microbial alterations contribute to aneurysm development or progression.

## Data Availability

The data presented in the study are deposited in the NCBI Sequence Read Archive (SRP723534) with BioProject accession number: PRJNA1506361.
