# Supplementary material for: Streptococcus and related oral taxa are differentially abundant in patients with abdominal aortic aneurysms compared with atherosclerotic disease controls
Source: Front Cardiovasc Med. 2026 Aug 28;13:1884994. doi: 10.3389/fcvm.2026.1884994 (PMC13562266; doi:10.3389/fcvm.2026.1884994)
Supplement: Supplementary file 1 [file Image1.pdf]

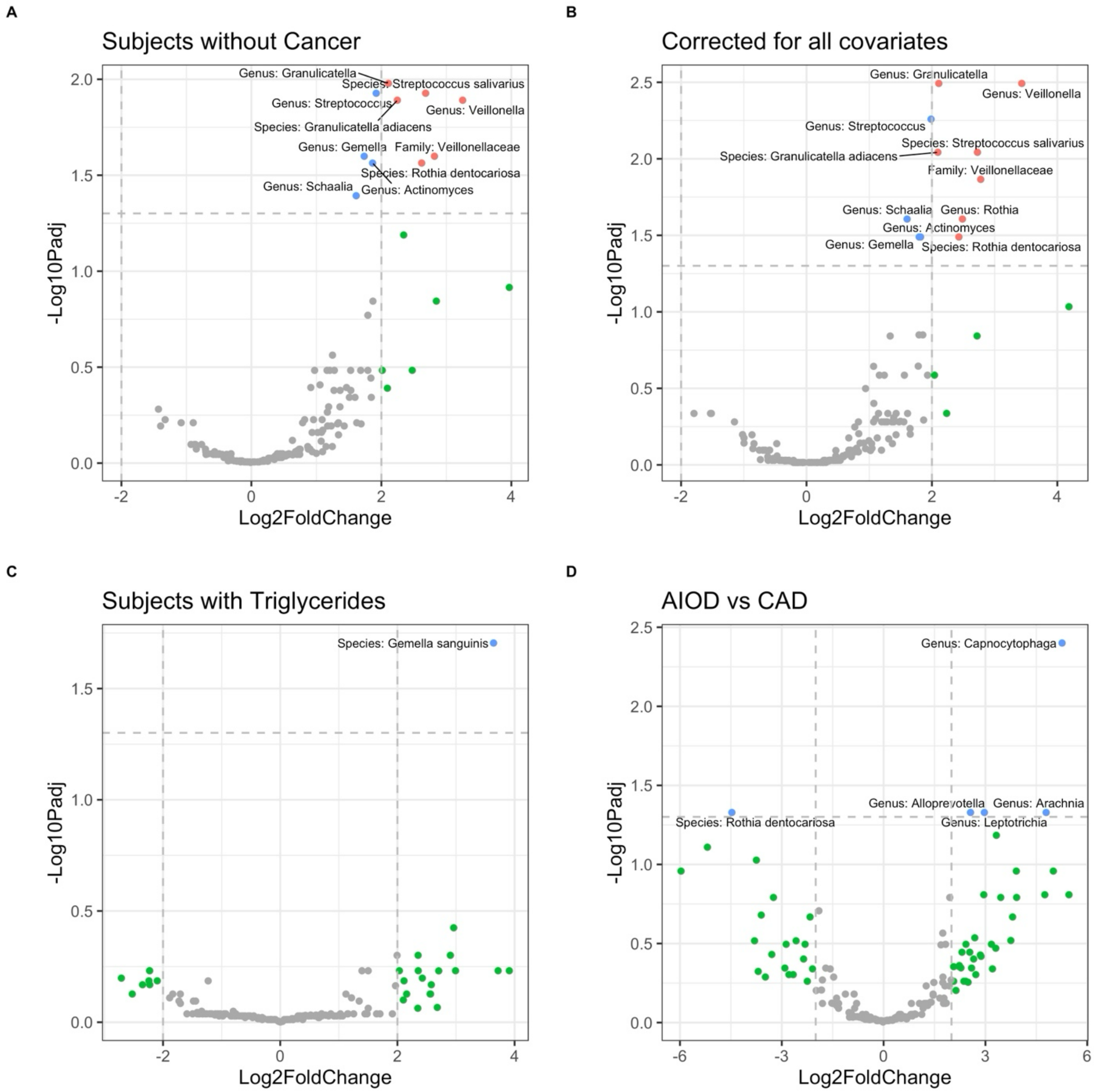

Supplemental Figure 1. Taxonomic Differential Abundance Sensitivity Analysis by Disease State. **A)** Volcano plot of differentially abundant bacterial species, genera, and families between patients with AAA and atherosclerotic controls using LinDA after all subjects with a known malignancy were removed. **B)** Volcano plot of differentially abundant bacterial species, genera, and families between patients with AAA and atherosclerotic controls using LinDA, with the model expanded to include diabetes, statin use, and PPI use. **C)** Volcano plot of differentially abundant bacterial species, genera, and families between patients with AAA and atherosclerotic controls using LinDA in a subset of 35 patients for whom blood triglyceride levels were available. Blood triglyceride level was included as a covariate in the model. **D)** Volcano plot of differentially abundant bacterial species, genera, and families between the atherosclerosis subcohorts of aortoiliac occlusive disease (AIOD) and coronary artery disease (CAD).
